# Perspectives of healthcare professionals on the pharmacist’s role in delivering vaccinations for patients with cancer: a qualitative study using role theory

**DOI:** 10.1007/s11096-025-01945-9

**Published:** 2025-06-16

**Authors:** Kristoffer Johnstone, Joyce Cooper, John Smithson, Beverley Glass

**Affiliations:** 1 James Cook University, Townsville City Campus, Townsville, Australia; 2 Pharmacy Department, Cairns Hospital, Cairns, QLD Australia; 3 School of Biomedical Sciences and Pharmacy, University of Newcastle, Newcastle, NSW Australia; 4 Faculty of Pharmacy, Rhodes University, Makhanda, South Africa; 5 School of Pharmacy, University of Jordan, Amman, Jordan

**Keywords:** Cancer, Expanded scope, Pharmacists, Role theory, Qualitative research, Vaccination

## Abstract

**Background:**

Vaccination rates for influenza and pneumococcal disease globally remain below targets for patients with cancer. Pharmacists already provide vaccinations in the community, highlighting an opportunity to bridge the gap by expanding hospital pharmacists scope of practice to deliver vaccinations in outpatient oncology settings.

**Aim:**

To explore the perceptions of multidisciplinary healthcare professionals (HCPs) for the potential expansion of pharmacists’ role to include a vaccination clinic for patients undergoing cancer treatment.

**Method:**

Semi-structured interviews were conducted with nurses, doctors, and pharmacists who were purposively sampled. Data analysis used a deductive coding approach, with data themed against the constructs of Role Theory: ambiguity, conflict, overload, identity, overqualification and underqualification.

**Results:**

Nineteen HCPs (6 nurses, 6 doctors, 7 pharmacists) participated. Participants identified the need for improved vaccination delivery, but considered this service not currently part of core business in the outpatient oncology setting. Role ambiguity was identified regarding which clinicians are ultimately responsible, with medical specialists inferring this to be the responsibility of primary care. Pharmacists’ identity was strongly linked to vaccination services in the community, but not in hospitals. HCPs thought oncology pharmacists had the knowledge to expand their role to deliver vaccination services. Pharmacists, while motivated, identified that adding responsibility could cause overload without appropriate resources.

**Conclusion:**

HCPs supported the inclusion of vaccination administration into the pharmacist’s role and highlighted that an appropriately vaccine-trained oncology pharmacist would improve access to vaccines for patients with cancer.

**Supplementary Information:**

The online version contains supplementary material available at 10.1007/s11096-025-01945-9.

## Impact statements

- Role definition and ownership of vaccine delivery are essential within the healthcare team to optimise vaccination rates for patients with cancer.
- Health care professionals agree that pharmacists within oncology settings have a role in providing vaccination services.
- Oncology pharmacists believe they have the knowledge of vaccines and cancer treatments but would need further training to be confident in their role to deliver a vaccine service.
- A pharmacist-led vaccination clinic in an outpatient oncology unit would require adequate resourcing to add this service to the core role of pharmacists providing cancer-specific pharmacy services.

## Introduction

Patients with cancer frequently have weakened immune systems, due to multiple factors such as inflammation, reduced bone marrow activity and immune-suppressing treatments [1, 2]. These patients, being more susceptible to infections, face a heightened risk of complications from vaccine-preventable diseases (VPDs) like influenza and pneumococcal infections. These infections can lead to severe consequences, including hospitalization and even death, impacting well-being and incurring healthcare costs [3].

Vaccination, the most effective strategy for preventing VPDs, serves a dual purpose of preventing infection and mitigating the severity of disease in cases where prevention has not been achieved. International and National guidelines, including the Australian Immunisation Handbook, recommend routine influenza and pneumococcal vaccination for all adults, while explicitly highlighting the need for vaccination in patients with cancer [4–6]. Despite these recommendations, vaccination rates in this patient cohort remain low [7] due to complex treatment schedules, concerns about vaccine safety and efficacy in immunocompromised individuals, and limited access to convenient vaccination services [8].

Non-live vaccines such as influenza and pneumococcal are usually safe, although their effectiveness may vary depending on the level of immunosuppression and timing of administration [9, 10]. As a result, vaccine strategies may differ for patients with cancer compared to the healthy general adult population [11]. Low vaccination rates in this cohort have highlighted the need for increased vaccination efforts, creating an opportunity for pharmacists to act by increasing access to vaccinations. The trend in high income countries, such as USA, UK, Canada, and Australia, has been to enable pharmacists’ autonomy in prescribing and administering vaccines [12]. A meta-analysis of pharmacist involvement in vaccination administration reported a positive impact on vaccination rates [13].

In recent decades, pharmacists’ roles have expanded from traditional dispensing and supply duties to working in close collaboration with other healthcare professionals in specialised roles and independent prescribing [14]. While their value in vaccinating the general public has been well-established and accepted [15], research is lacking on their role in vaccinating high-risk patients, such as patients with cancer undergoing treatment. Pharmacist-led clinics have demonstrated effectiveness in increasing vaccination rates within other vulnerable or high-risk populations such as the elderly, or school children with the administration of human papilloma virus (HPV) [12, 13, 16]. These clinics have leveraged pharmacists’ expertise in medication management, patient education, and immunisation to provide accessible and efficient vaccination services [16].

This study employs a Role Theory framework to understand how healthcare professionals perceive the role of pharmacists in providing vaccination services for patients with cancer. This theory was employed to inform how previous experiences with pharmacists and pharmacy services would impact health professionals’ perspectives of a proposed expanded role for pharmacists in the hospital oncology setting and how this aligns with their own professional role in caring for patients with cancer [17, 18].

### Aim

The aim of our qualitative study was to explore the perceptions of multidisciplinary healthcare professional (HCP) team of the potential expansion of pharmacists’ role to include a vaccination clinic for patients undergoing cancer treatment.

### Ethics approval

Ethics approval has been granted from Far North Queensland ethics committee HREC / /2023/QCH69788 (Apr Ver 3) – 1628.

## Method

This study followed a qualitative exploratory design, with data collected using semi-structured interviews, to explore health professionals’ perspectives of pharmacists’ role in vaccination delivery and Role Theory for data analysis and discussion [17]. Interview guide can be found in supplementary material.

To ensure a comprehensive reporting of the study’s findings, the Consolidated Criteria for Reporting Qualitative Research (COREQ) guidelines was followed [19].

### Study setting and sampling

This study was undertaken in the oncology outpatient unit of Cairns Hospital, a large regional hospital in Australia. We employed a purposive and subsequent snowballing sampling approach for interview recruitment [20–22]. Email invitations were sent to pharmacists, nurses, and doctors who had worked in the oncology outpatient unit. Participating staff were encouraged to refer colleagues after the interview without disclosing the interview questions. All participants were required to be directly involved in patient care in an oncology setting, within the past 3 months. Enrolment continued until data saturation was achieved during the interviews with no new themes emerging.

### Theoretical framework

Role theory framework guiding the analysis of the resulting data [17, 23], aligns with previous pharmacy research into development and implementation of pharmacy services. Role theory posits that individuals within a system occupy distinct social positions, each with a set of expectations, such as in a hospital setting, where nurses, doctors, patients and pharmacists all have a defined role [17, 24]. This allowed us to identify potential areas of conflict and opportunities for enhanced collaboration [18, 25]. Definitions were developed for this study based on existing literature in pharmacy and nursing research, with these definitions adapted from the work of Brookes et al., Guirguis et al., and Taylor et al., as outlined in Table 1 [17, 18, 24, 26].

**Table 1:** Definition of Role Theory [17, 18, 24, 26]

Role theory construct	Definition
Role ambiguity	Disagreement on role expectation associated with a lack of clarity of those expectations
Role identity	Individual’s interpretation of role expectation, that is, position-specific norms identifying the attitudes, behaviours and cognitions required and anticipated for a role occupant
Role conflict	Focal person perceives existing role expectations as being contradictory or mutually exclusive
Role overload	Too many role expectations for the role occupant to complete in the time given and resources
Role underqualification	Individual requires more training to successfully perform the given role
Role overqualification	Individual's role expectations are less than their education qualifies them to accomplish

### Data collection

Given the exploratory nature of this study, to gain in-depth insights into vaccination practices and the role of pharmacists vaccinating patients with cancer, a qualitative approach utilising semi-structured interviews was chosen [27]. Designed with the Diffusion of Innovation Theory (DOI) as the framework [28, 29], the interview guide explored HCPs perceptions on the implementation of a pharmacist-led vaccination clinic for patients with cancer. The interview guide incorporated open-ended questions, and follow-up prompts resulting in a rich dataset. Participants during then interviews revealed and in fact emphasised the role and integration of the pharmacists within the healthcare system. To take advantage of the richness if the data and to capture the nuances of these perspectives, a separate thematic analysis was conducted guided by the constructs of Role Theory [17], which provided a more pertinent lens with a focus on the specific roles and responsibilities of hospital pharmacists to deliver vaccinations and improve vaccination rates in patients with cancer [30–32].

The primary researcher conducted all interviews between February and May 2024, either in-person or via Microsoft Teams. Participants provided informed consent prior to the interview, and all identifying information besides their profession was removed during the transcription process.

### Data analysis

All interviews were digitally recorded, transcribed verbatim and transcripts de-identified. NVivo ™ software was used for the thematic analysis of the interview data. This iterative process involved several stages: Familiarisation with data, initial coding, theme development, theme review and identifying illustrative quotes [33, 34]. During the coding of data against the constructs of the diffusion of innovation theory [28], the perceived role of the pharmacist emerged as a crucial aspect of the participants experience with the vaccination of cancer patient in a hospital setting.

The analysis then adopted a deductive coding, utilising a simplified framework inspired in 1988 by Hardy and Conway [17]. The six established themes of role theory; ambiguity, conflict, overload, identity, role overqualification and role underqualification served as the predetermined codes for analysing the transcripts. Secondary inductive coding was used for refining analysis to determine whether there were any additional themes [35].

To ensure rigour of our findings, primary author (KJ) conducted thematic analysis on three transcripts, representing the perspectives of a doctor, nurse, and pharmacist. These transcripts were subsequently coded for consistency by BG and JC and any discrepancies were resolved by discussion with all authors until consensus was reached [36]. Verbatim quotes were used to strengthen the credibility of the findings, and all team members were involved in the analysis process to ensure interpretations were grounded in the data rather than any preconceived notions [37].

### Reflexivity

The research team comprised of KJ, JC, JS and BG, experienced in qualitative pharmacy research. Reflexivity was central, involving conscious awareness and examination of our own biases. Through reflexive practices, peer review, and critical self-reflection, we aimed to enhance the credibility. Given our diverse experience including in hospital pharmacy (JC), cancer unit staff member (KJ), and community pharmacy vaccination pilots (BG), a multi-step analysis was employed to mitigate bias. A supervised pilot interview was conducted to identify potential bias and refine the interview questions [38].

## Results

Nineteen interviews were conducted with HCPs including six doctors (2 haematologists and 4 medical oncologists), seven pharmacists, and six nurses. Fifteen interviews were held in-person and four were conducted virtually using Microsoft Teams™. Interviews ranged from nine to twenty-six minutes, with an average duration of twelve minutes. Participant characteristics are summarised in Table 2.

**Table 2:** Participants’ characteristics

	Pharmacist	Nurse	Doctor
	N = 7	N = 6	N = 6
*Gender*			
Male	1	–	3
Female	6	6	3
*Years of professional practice experience*			
< 5	1	–	–
06-10	2	1	–
11-15	2	–	3
16–20	2	1	1
21–25	–	2	1
26–30	–	1	-
30 +	–	1	1
*Years of experience in oncology setting*			
< 5	3	1	–
06-10	3		3
11-15	1	4	2
16–20	–	1	–
21–25	–	–	–
26–30	–	–	1
> 30	–	–	–

Participants, although asked about the potential implementation of a pharmacist-led vaccination clinic often made reference to the role of the pharmacist in this clinic with the emerging themes mapped against the constructs of Role Theory as shown in Table 1 [17] and quotes assigned to doctors (D), pharmacists (P) and nurses (N) respectively.

### Role ambiguity

Participants reflected on the following two aspects: Who’s role is it to be ultimately responsible for vaccinating patients, and what is the pharmacist’s role in a cancer unit regarding vaccination.

Doctors, who were all specialist oncologists or haematologists, reflected on their role when discussing vaccination stating that they relied on General Practitioners (GPs) to be responsible for primary health, and thus a patient’s vaccination needs. Specialists had minimal time with patients and prioritised discussing their cancer treatment. They believed that GPs are ultimately responsible for patient’s preventative health needs, but acknowledged that it is not done well, while patients are undergoing treatment for cancer.

“GPs think cancer services will be doing it, cancer service thinks GP will be doing it. The responsibility for who it sits with is often not clear” (D6)

There were comments that having multiple healthcare professionals involved in vaccination could cause confusion between specialists, GPs, and pharmacists, as to who is responsible for vaccination.

“Who is overseeing the vaccinations, who is prescribing, who is following up? “(D5)

There was some acknowledgement that vaccine administration is still perceived to be part of the nurse’s core role and identity, but the recent changes in pharmacists providing vaccination services in community pharmacies have caused some discussion around who should perform this role.

“Absolutely it sits better with nurses because they’re familiar with IM [intramuscular] administration and do it all the time. But pharmacists also do it all the time, just something we haven’t been exposed to as a hospital pharmacist” (P3)

### Role identity

The role of community pharmacists in providing vaccination services has changed significantly over the past several years, especially since COVID-19 [39]. Many participants identified that community pharmacists are commonly vaccinating the public and this is now a recognised role for pharmacists, with many acknowledging that they got their own flu and COVID vaccination from community pharmacies.

“In terms of pharmacy, you guys [pharmacists] outside of hospital are doing COVID and flu vaccine anyway” (D2)

“If you are resourced to, I think pharmacists would be better than particularly a nurse, because of the knowledge of medications and when a good time to get that injection” (D1)

Interviews revealed that the HCPs perceived verifying patient vaccination history as a low priority within the cancer unit. They explained that they lacked time to conduct these assessments and did not consider it a primary role of a HCP within the unit.

“In terms of my own practice and experience, I guess discussing vaccination for influenza and pneumococcal is not something I tend to actively think about or prioritise” (D3)

### Role conflict

Traditionally, there has been a clear division of clinical roles between doctors and pharmacists [40]. However with some apparent overlap in services, skills and increasing expanded scope of practice, there is potential for conflict or competition between professions [41]. Our participants perceived the conflict between professions to be minimal and acknowledged that oncology trained pharmacists possess the necessary skills to manage vaccines. However, doctors still felt the need to retain overall decision-making authority regarding vaccinations for their patients.

“They’re [patients] at higher risk that needs more shared decision making. And there are some practitioners that are quite prescriptive about the who and when for things like flu around timing it around chemotherapy. But I acknowledge that for the majority for influenza and COVID and pneumococcal doesn’t need higher input” (D6)

With the increasing involvement of pharmacists in vaccine administration, there may be potential for role conflict with nurses, who have historically played a significant role in vaccinations [42]. However, in our study, nurses believed that pharmacists with their specialised drug knowledge and experience managing post-transplant vaccination schedules, were well equipped to handle vaccines for patients undergoing cancer treatment.

“I am very multi-disciplinary in my nursing, so I am very open to each individual role. I feel like you guys [pharmacists] know more about vaccines than I do.” (N5)

There was also acknowledgement of the progress pharmacists have made in expanding their roles into immunisation of the general population and the improved acceptance of pharmacists undertaking these tasks.

“I think if this had been brought up, maybe 5 years ago, there might have been some stepping on toes” (P2)

### Role overload

Most of the HCPs acknowledged that a pharmacist-led vaccine clinic could impact the pharmacist’s role and cause a work overload to the pharmacy team. Capturing all the patients with cancer that are eligible for vaccination, even for just influenza and pneumococcal, was thought to be above the current capacity of the oncology pharmacists to meet the demand of the service.

“there would have to be a change in the way we do things for freeing up of time. You know, within the current workload” (D5)

Doctors and pharmacists raised the issue that pharmacists are capable of vaccinating, but held concerns for impact on “core” pharmacist business of cancer care, which involves seeing patients, clinically verifying cancer treatment and procuring chemotherapy. Pharmacists play crucial roles in oncology units and doctors and pharmacists felt that vaccine services was not part of that current core pharmacist role in a cancer unit and would be challenging to achieve within existing resource allocation.

“As in terms of in-house [pharmacist] vaccination, I guess initially I don’t have concerns, I can’t think of anyone who would concerns about this unless it was impacting on our ability to do our core business.” (D3)

### Role underqualification

Participants indicated that there is a lack of clarity regarding the specific qualifications required for pharmacists to administer vaccinations, who has these qualifications and how to identify them. A notable distinction was observed between hospital and community pharmacists, reflecting a lack of understanding of the scope of vaccination services approved for pharmacists in each setting. Despite many hospital pharmacists being qualified to give vaccinations from undergraduate and post graduate training, these skills were not utilised within the hospital setting.

“When you start looking at branching out and extending your [pharmacist] scope of practice, you’re going to have to be really clear and make sure you meet with all stakeholders” (N6)

Pharmacists had some concerns about the skills required in delivering the service with their current experience and training.

“Upskilling of certain people to actually administer vaccines, we need to upskill” (P3)

There were more concerns around the skills and ability of pharmacists to respond to adverse reaction reactions and anaphylaxis, when administering vaccines.

“could definitely try and get pharmacists administer IM (intra-muscular) medications, it would be the aftermath of that. The waiting for 15 minutes and monitoring for side effects and allergic reactions and how pharmacists then deal with the allergic reaction.” (N5)

### Role overqualification

There was negligible mention of pharmacists being overqualified to provide vaccinations or this being a poor use of their time and skills. Most participants felt that this has become the standard role of pharmacists, with the appropriate qualifications. Some nurses however felt that pharmacists had other skills that could be utilised and that the task of vaccine administration could be left to them. The nurses felt pharmacists could take responsibility of assessing the patients, scheduling, and prescribing vaccines and then nurses would administer the vaccines.

“I think it rolls into your day-to-day MOSAIQ (electronic oncology prescribing software) scheduling. I think it would add an extra dynamic to your pharmacy unit and the nurses give the injections day to day.” (N5)

## Discussion

### Statement of key findings

This study has explored the perspectives of key stakeholders on the role of pharmacists in administering vaccination to patients with cancer in an oncology setting at a regional Australian hospital. Vaccination rates in patients with cancer have been identified to be substandard worldwide [7, 43, 44], and this study identified that although vaccination in patients being treated for cancer is considered part of primary healthcare and the responsibility of GPs, it is acknowledged that this is not often achieved. Oncologists and haematologists indicated that GPs were not confident with vaccinating patients undergoing treatment for cancer without prior approval from a treating specialist, causing significant delays to appropriate vaccinations. Doctors and nurses working in the oncology unit, thought pharmacists had the most up to date knowledge on treatments, immunisation guidelines and could deliver timely vaccinations for patients receiving cancer treatment feeling comfortable for pharmacists to take ownership of vaccination in this setting.

Our study highlighted that there are different perspectives of the roles of community-based pharmacists and hospital-based oncology pharmacists in respect to delivering vaccination services. Doctors and nurses assumed that there was different training and qualifications for the two areas of pharmacy practice. Participants thought that oncology pharmacists had the knowledge of vaccines, but were unsure of their level of training or whether they had the skills for administering vaccines and managing potential anaphylactic reactions. All healthcare professionals acknowledged that pharmacists could have an impact on vaccination rates, but are currently not adequately resourced to offer this service.

### Strengths and limitations

To our knowledge, this is the first qualitative study examining the pharmacist’s role in vaccination in an oncology setting from the perspectives of healthcare professionals. Including perspectives from healthcare professionals with varying degree of experience in a cancer unit the strengthened our findings. Strong methodology with coding on a well-established framework with Role Theory, independent coding review and discussion, and pilot interview improved consistency and removed perceived bias and increase trustworthiness of the results.

Limitations included potential volunteer selection bias from a single site with a snowballing recruitment method, limiting the generalisability of the findings. Despite data saturation achieved, a larger sample size and multiple sites would be beneficial. Prior knowledge of the primary researcher might have influenced responses, leading to an overestimate of positive outcomes [45]. However, this was minimised by having structured questions, and ongoing reflexivity. Additionally, participants who volunteered to participate may have had strong opinions to share, which could have influenced the data.

### Interpretation

The perspectives of healthcare professionals on pharmacists vaccinating patients with cancer are shaped by personal and professional experiences of both hospital and community pharmacists and pharmacy services [24, 46]. Pharmacy is adapting to meet the increasing health demands of an aging population, which has included primary health screening, vaccine services, medication safety and prescribing [47]. Majority of HCPs suggested that the community pharmacist’s role is clear in vaccination, but not so for hospital pharmacists. This is most likely associated with lack of previous pharmacist-led vaccine services in hospitals [48], with most vaccine clinics run within Australian public hospitals being nurse-led [49]. Our study’s findings are further supported by a systematic review that identified positive perceptions from other healthcare professionals of hospital pharmacy services in areas such as medication governance, medication reconciliation, drug information and safety, but limited recognition of pharmacist’s involvement in immunisation services [50].

Our study found that while healthcare professionals recognised the importance of vaccination status for patients with cancer, it wasn’t a top priority in the outpatient setting. Challenges identified of ensuring appropriate vaccination for patients starting cancer treatment included time constraints, deficient medical records, and unclear vaccine requirements. Despite guidelines recommending vaccination with influenza and pneumococcal for all patients and their close contacts [51], our study highlighted that there is a lack of ownership for vaccination of patients in the oncology and haematology treatment settings. There was role ambiguity in determining who was ultimately responsible for ensuring patients would be routinely vaccinated. With the haematologists and oncologists’ reliance on GPs to provide vaccines, they acknowledged that they are most likely not up to date with a patient’s vaccination status, however this is confounded by the GPs reluctance to vaccinate without specialist approval. Literature supports our participants perspectives with GPs shown not to be comfortable in providing vaccines to patients with cancer due to inconsistent recommendations on benefits or safety [52]. This study identified potential shortcomings in communication between cancer care providers and GPs. Notably, there was evidence of limited two-way communication regarding patients’ current treatment and vaccination status. This lack of information exchange could potentially hinder optimal patient care. Effective communication between healthcare professionals was found to be crucial for ensuring continuity of care and reducing the risk of medication errors or missed preventative measures such as vaccinations [53].

Study participants supported hospital pharmacists assuming a vaccination role within the oncology setting. Participants recognised pharmacists appropriate clinical skills and judgement to vaccinate appropriately and accurately document. Patients with cancer, face multiple barriers to receive appropriate vaccination. Pharmacists in outpatient oncology units could address these barriers by providing patient education, counselling on vaccine safety and effectiveness during treatment in the absence of other healthcare professional recommendations [54].

Study findings correlate with other pharmacist vaccination programs, where many pharmacists expressed willingness to vaccinate [55]. All healthcare professionals emphasised the need for appropriate training and credentialing for pharmacists to provide vaccine services and handle adverse events. This aligns with other studies, which found similar potential barriers to implementation of pharmacy vaccine services [56, 57].

Pharmacists in this study expressed concerns about additional workload associated with expanded services. While acknowledging their clinical capabilities, they emphasised the need for additional resources. Studies across the globe highlight the potential benefit of expanding pharmacists’ roles, but also emphasise the importance of careful implementation [58, 59]. A study with Canadian pharmacists suggested creating a detailed plan to implement expanded practice services to reduce potential of overload [60]. Cairns Hospital, a large regional hospital in northeast Australia that serves a diverse population with complex needs. Located in a regional area, it faces staff shortages and limited resources compared to metropolitan centres. This must be considered when introducing additional roles to a potentially overburdened pharmacy workforce. Actions to overcome barriers to implement pharmacist vaccination services should include; program co-design that includes input from all stake holders, emphasises pharmacist training and qualifications and previous successful examples of pharmacist-led programs, advocate for increasing pharmacist numbers to achieve better staff-to-patient rations, streamlining workflows to improve efficiencies and reduce burden on staff and incentivise pharmacist participation with some form of remuneration.

## Further research

Future research should explore the generalisability of these findings by including a larger sample size across multiple cancer centres. Investigating the perspectives of consumers and primary care physicians of vaccination responsibilities would also provide a more comprehensive understanding of the current landscape. Additionally, implementing a pilot program to expand pharmacists’ roles in cancer units to include vaccinations is warranted to evaluate the impact on vaccination rates and patient outcomes.

## Conclusion

Despite recognising the role of community pharmacists in vaccination services, HCPs lacked a clear understanding of the specific roles and capabilities of hospital pharmacists in providing vaccination services to patients with cancer. This uncertainty, coupled with time constraints for specialists, contributes to a reliance on GPs, who may feel ill-equipped to vaccinate patients undergoing cancer treatment. Our findings suggest that hospital pharmacists, with their existing expertise, could play a role in addressing this gap and improving vaccination rates among patients with cancer.

## Supplementary Information

Supplementary file1 (DOCX 5 kb)
