# Supplementary material for: Perspectives of healthcare professionals on the pharmacist’s role in delivering vaccinations for patients with cancer: a qualitative study using role theory
Source: Int J Clin Pharm. 2025 Jun 16;47(6):1760–9. doi: 10.1007/s11096-025-01945-9 (PMC12630266; doi:10.1007/s11096-025-01945-9)
Supplement: Supplementary file 1 — Supplementary file1 (DOCX 5 kb) [file 11096_2025_1945_MOESM1_ESM.docx]

**Appendix A**: Consolidated Criteria for Reporting Qualitative research (COREQ)

|  |  |
| --- | --- |
| 1. Interviewer/facilitator | KJ- first author |
| 2. Credentials | BPharm, Graduate Diploma in Public Health and Tropical Medicine, FANZCAP, MPhil (Health) candidate |
| 3. Occupation | Pharmacist |
| 4. Gender | Male |
| 5. Experience and training | Over 10 years of cancer experience, post graduate studies and undertaking higher research degree |
| 6. Relationship established | Yes - colleagues |
| 7. Participant knowledge of the interviewer | Colleagues- work in the same unit and knew research was associated with MPhil degree |
| 8. Interviewer characteristics | Bias – as part of the interview schedule it was discussed that these views are their own and should not be influenced by what they thought the interviewer would want to hear.  As a pharmacist, it could be perceived of wanting to have a positive influence on pharmacists expanded roles. |
| 9. Methodological orientation and  Theory | Interview guide= Diffusion of Innovation Theory  Analysis= Role Theory for thematic analysis |
| 10. Sampling How were participants selected? | Purposive sampling and snowballing |
| 11. Method of approach | Direct and broad email recruitment to department along with presentations at discipline specific meetings of nurses, doctors and pharmacists. |
| 13. Non-participation | Not accounted for |
| 14. Setting of data collection | Hospital clinic room or via Microsoft teams virtually |
| 15. Presence of non-participants | No one else present during interviews, all interviews were recorded. |
| 16. Description of sample | There was wide range of experience in most professions, ranging from a few years to over 30 years. It was a general representative of the gender make up of health professionals with even split of male and female doctors and more female pharmacists and nurses. |
| 17. Interview guide | Interview guide is provided with introduction statement, questions and follow up prompts.  The interview schedule was piloted and reviewed by senior researcher. |
| 18. Repeat interviews | N/A |
| 19. Audio/visual recording | Yes- the interviews were audio recorded when conducted face to face and online interviews via Microsoft teams ™ were audio recorded and transcribed. |
| 20. Field notes | Minimal field notes were used |
| 21. Duration | The interviews ranged from 9 minutes to 26 minutes with an average of 12 minutes |
| 22. Data saturation | Data saturation is discussed when no new themes emerged |
| 23. Transcripts returned | No- all transcripts were clear in recording and no clarification required. |
| 24. Number of data coders | 1 primary coder with 2 secondary coders to check for consistency |
| 25. Description of the coding tree | No |
| 26. Derivation of themes | Themes were identified in the literature in advance and coded to a known framework |
| 27. Software | NVIVO ™ was used for analysis |
| 28. Participant checking | No |
| 29. Quotations presented | Quotes have been provided for all themes |
| 30. Data and findings consistent | There were consistent findings in the data for most themes but some conflicting results which gave a good overall presentation of the data and alternative views. |
| 31. Clarity of major themes | Major themes have been presented in the results for the 7 constructs of role theory. |
| 32. Clarity of minor themes | Minor themes have not been coded due to the word limit restrictions and grouped into major themes. |
